# High dose dietary vitamin D allocates surplus calories to muscle and growth instead of fat via modulation of myostatin and leptin signaling

**DOI:** 10.21203/rs.3.rs-4202165/v1

**Published:** 2024-05-08

**Authors:** Jeffrey Roizen, Caela Long, Alex Casella, Michael Nguyen, Lo Danahy, Christoph Seiler, Meizan Lei, Julian Mark

**Affiliations:** Childrens Hosp Philadelphia, Div Endocrinol & Diabet, 34th & Civ Ctr Blvd, Philadelphia, PA 19104 USA; The Children’s Hospital of Philadelphia and The University of Pennsylvania Perelman School of Medicine.; The Children’s Hospital of Philadelphia and The University of Pennsylvania Perelman School of Medicine.; The Children’s Hospital of Philadelphia and The University of Pennsylvania Perelman School of Medicine.; The Children’s Hospital of Philadelphia and The University of Pennsylvania Perelman School of Medicine.; Children’s Hospital of Philadelphia; The Children’s Hospital of Philadelphia and The University of Pennsylvania Perelman School of Medicine.; The Children’s Hospital of Philadelphia and The University of Pennsylvania Perelman School of Medicine.

**Keywords:** vitamin D, myostatin, leptin, energy sensing, calorie allocation, growth, seasonal, metabolism

## Abstract

Obesity occurs because the body stores surplus calories as fat rather than as muscle. Fat secretes a hormone, leptin, that modulates energy balance at the brain. Changes in fat mass are mirrored by changes in serum leptin. Elevated leptin prompts the brain to decrease appetite and increase energy expenditure. In obesity, however, impaired leptin sensitivity mutes these leptin-mediated changes. We have limited understanding of what controls leptin production by fat or leptin sensitivity in the brain. Muscle produces a hormone, myostatin, that plays a role in muscle analogous to the one that leptin plays in fat. Absent myostatin leads to increased muscle mass and strength. As with leptin, we also do not know what controls myostatin production or sensitivity. Although fat mass and muscle mass are closely linked, the interplay between leptin and myostatin remains obscure. Here we describe an interplay linked thru vitamin D. Conventionally, it is thought that vitamin D improves strength via trophic effects at the muscle. However, we find here that high dose dietary vitamin D allocates excess calories to muscle and linear growth instead of storage as fat. Vitamin D mediates this allocation by decreasing myostatin production and increasing leptin production and sensitivity. That is, high dose vitamin D improves integration of organismal energy balance. Obesity, aging and other chronic inflammatory diseases are associated with increased fat mass and decreased muscle mass and function (e.g. sarcopenia). Our work provides a physiologic framework for how high-dose vitamin D would increase allocation of calories to muscle instead of fat in these pathologies. Additionally, our work reveals a novel link between the myostatin and leptin signaling whereby myostatin conveys energy needs to modulate leptin effects on calorie allocation. This result provides evidence to update the conventional model of energy stores sensing to a new model of energy balance sensing. In our proposed model, integration of leptin and myostatin signaling allows control of body composition independent of weight. Furthermore, our work reveals how physiologic seasonal variation in vitamin D may be important in controlling season-specific metabolism and calorie allocation to fat in winter and muscle and growth in summer.

## INTRODUCTION

Vitamin D signaling has been implicated in conveying nutrient status to the brain^1^. Vitamin D signaling is mediated primarily by activation of the vitamin D receptor (VDR)^2^. VDR knockout mice have a complex “failure-to-thrive” phenotype with abnormal serum calcium and phosphate, poor growth and nearly-absent white fat^3^. Their failure to thrive occurs, in part, because they have decreased storage of lipids into white fat^3–6^. White fat produces the majority of circulating leptin. Thus, the lack of white fat in VDR knockout mice causes them to have persistently low leptin^3–6^.

In addition to modulation of leptin secretion and nutrient sensing, vitamin D signaling also plays a role in muscle function. Low vitamin D has long been known to cause muscle weakness that is relieved by replenishment of vitamin D. Animal models to date have primarily examined vitamin D signaling and effects through experimental models of deficiency. Specifically, investigators have used approaches focusing on limitation of dietary vitamin D, or conventional or tissue specific knockout of the VDR^7–11^. A number of clinical studies, however, suggest that increasing vitamin D within the normal range may have additional beneficial effects on muscle function^12–16^.

Scientists have largely examined these functions of vitamin D, regulation of fat metabolism and regulation of muscle mass and function, separately. However, fat mass and muscle mass are closely linked. Interventions that increase muscle mass increase both weight and fat mass. Conversely, when weight loss exceeds 10% of baseline weight, muscle mass loss increases proportionally to fat mass loss suggesting a complex relationship between fat and muscle mass. The mechanisms linking fat mass and muscle mass are poorly understood. The loss of muscle mass once weight loss exceeds 10% suggests that another checkpoint in energy balance may be energy needs. Myostatin has been conceptualized as having homeostatic effects on muscle mass. Our data suggests that myostatin may also work to convey energy needs.

Conventional VDR knockout mice have very low serum leptin and muscle specific VDR knockout mice have excess production of myostatin mRNA^3,8^. We sought to examine the relationship between vitamin D signaling, fat mass, muscle mass, and muscle function. Further, we wanted to examine this relationship not only by comparing normal-to-low dietary vitamin D but also by comparing normal-to-high dietary vitamin D.

To evaluate the hypothesis that high-dose dietary vitamin D provides muscle function benefit *beyond* normal-dietary vitamin D, we sought to determine the effect of increasing vitamin D within the normal range (from 20–30 ng/dL to above 30 ng/dL) improved muscle function in adult wild-type mice. Normal dietary vitamin D increased strength over low-dose vitamin D without altering lean mass. High-dose dietary vitamin D significantly improved strength beyond normal vitamin D. Moreover, high-dose vitamin D also increased lean mass without affecting weight. That is, high-dose vitamin D effectively redistributed calories from fat to muscle.

Replenishing vitamin D from low-to-normal decreased serum myostatin and increased the amount of leptin produced per fat mass. High-dose vitamin D increased sensitivity to leptin without significantly affecting the amount of leptin produced per fat mass. This increased leptin sensitivity did not alter appetite but did increase energy expenditure. High-dose vitamin D also increased linear growth and lean mass proportion of weight in mice. Mendelian randomization revealed that vitamin D increases linear growth in humans, confirming the clinical importance of these findings. We also found that high-dose vitamin D improved the length of early zebrafish, supporting the evolutionary significance of our findings.

**Figure 1:**
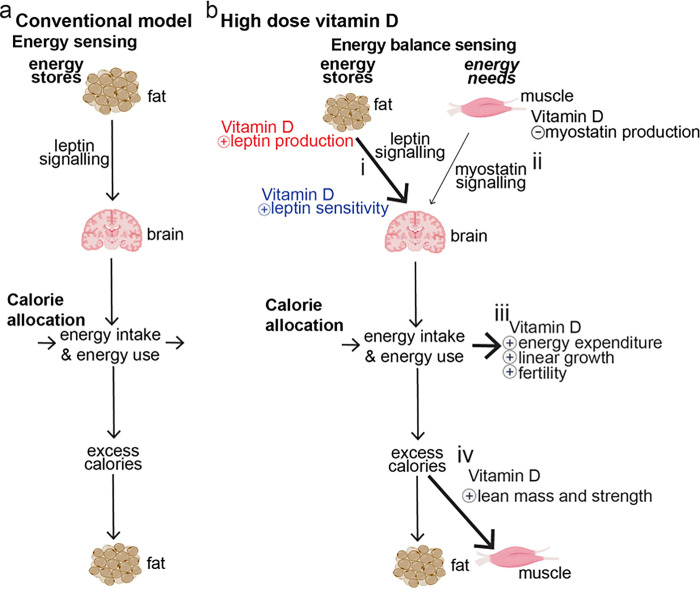
Visual Abstract: High dose dietary vitamin D preferentially allocates excess calories to muscle and growth instead of fat by increasing leptin production and sensitivity and decreasing myostatin production. **a)** In the Conventional Model (left side) energy stores sensing is mediated by leptin and by default excess calories are stored as fat. **b)** in **High dose Vitamin D** (**b**, at bottom)). Our results below reveal a role for vitamin D in modulating energy balance sensing as well as calorie allocation. **i)**Vitamin D increases (+) leptin production and sensitivity (overall increasing leptin signaling (bold arrow)), and **ii.)** vitamin D decreases myostatin production (decreasing myostatin signaling (thin arrow)), leading to **iii)**increased energy expenditure, linear growth and improved fertility as well as **iv)** increased allocation of excess calories to muscle (bold arrow)

Thus, here we report for the first time that high dose dietary vitamin D preferentially allocates excess calories to muscle and growth instead of storing them as fat by decreasing myostatin signaling and increasing leptin production and sensitivity. This result provides evidence to update the conventional model of energy sensing (Fig. 1a) to a new model of energy balance sensing (Fig. 1b) where integration of leptin and myostatin signaling allows control of calorie allocation.

## RESULTS

### High dose vitamin D significantly improves grip strength compared to normal vitamin D

**Figure 2:**
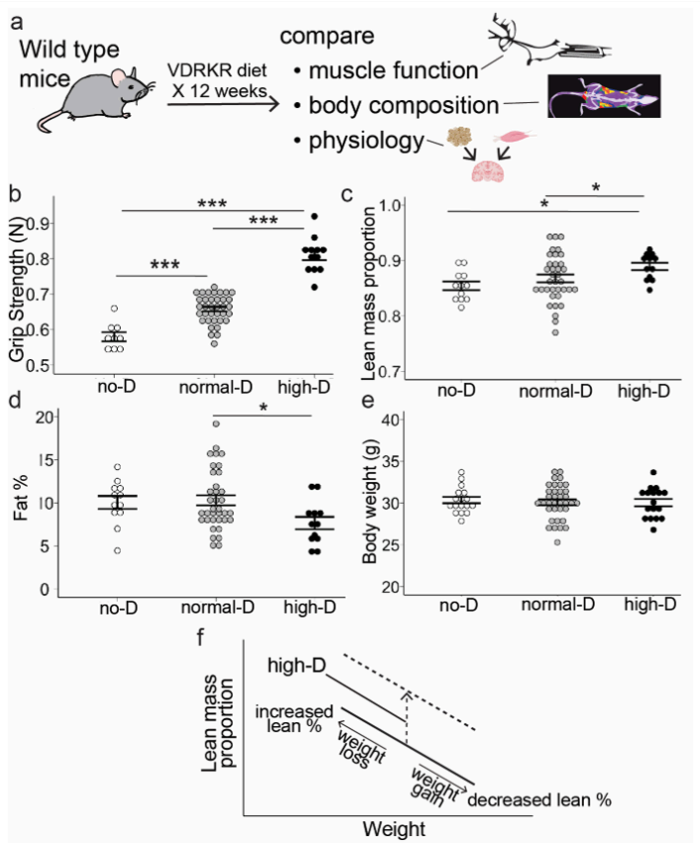
High dose vitamin D builds muscle without altering weight. **a.** Wild-type mice were maintained on a defined vitamin D receptor knockout rescue (VDRKR) diet with each of different doses of vitamin D for 12 weeks and then the effects of each dose of vitamin D on strength, body composition and physiology were assessed. **b.** High-D improves grip strength (*** 0.001>p for each by Tukey post-tests) **c.** High-D improves lean mass (*= p<0.05 for each by Tukey post-tests). **d.** High-D decreases fat mass (*= p<0.05 for each by Tukey post-tests). **e.** High-D does not alter body weight. **F.** High-D shifts the curve of the relationship between weight and lean mass. In the linear relationship between lean mass and weight: weight gain decreases the proportion of lean mass and weight loss increases the proportion of lean mass. High-D shifts this curve vertically such that lean mass proportion increases for the same weight.

To examine calciometabolic-independent effects of vitamin D on muscle, we used the vitamin D receptor knockout rescue diet (VDRKR diet) and varied vitamin D in wild-type (C57BL/6) male mice. We used three levels of added vitamin D in the diet: 0 IU/kg (no-D), 2000 IU/kg (normal-D) and 10,000 IU/kg (high-D). After 4 weeks, these diets allowed us to achieve target serum 25(OH)D of less than 5 ng/mL, between 20–30 ng/mL and above 30 ng/mL (experimental schematic in Fig. 2a).

To determine whether high-D improves muscle function in a dose-responsive fashion above the bottom of the normal range, we measured grip strength as previously described^17^. Normal-D significantly improved grip strength over no-D. High-D more dramatically increased grip strength over normal-D (Fig. 2b, ***p < 0.001 for each by Tukey’s Post-hoc test). *While the improvement in going from no-D to normal-D is expected, the more substantial improvement in going from normal-D to high-D is novel*.

### High dose vitamin D increases lean mass, but normal vitamin D does not

To examine whether the high-dose vitamin D stimulated increase in strength reflected changes in muscle mass, we measured mouse body composition via NMR as described previously^17^. Normal-D did not increase lean mass over low-D but high-D increased lean mass and decreased fat mass relative to normal-D *without altering weight* (Fig. 2c for lean mass, *: p < 0.05 by ANOVA with Tukey post-tests; Fig. 2d for fat mass, *: p < 0.05 by ANOVA with Tukey post-tests; Fig. 2e for weight, p > 0.05 by ANOVA). Thus, the high-dose vitamin D stimulated increase in strength is mediated in part by increased calorie allocation to build muscle mass instead of fat mass (physiologic schematic in Fig. 2f).

### Increased vitamin D inhibits myostatin production

**Figure 3:**
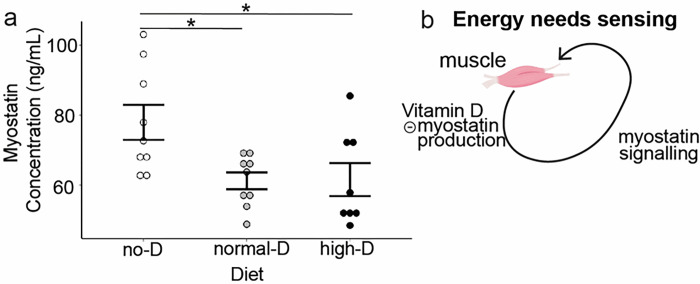
Increasing vitamin D inhibits myostatin production. Wild-type mice were maintained on defined CHOW diets with different doses of vitamin D. At sacrifice serum was harvested and myostatin was measured by ELISA. **a.** Normal-D and high-D decrease myostatin concentrations vs no-D (*= p<0.05, for each ANOVA with Tukey post-tests). **b.**Normal-D and high-D inhibit myostatin production.

Recent work in muscle-specific VDR knockout mice as well as in mouse models of vitamin D deficiency reveal that normal vitamin D concentrations facilitate VDR-dependent decreases in myostatin mRNA in muscle^11^. To examine the possibility that vitamin D dose alters circulating myostatin to regulate the proportion of muscle mass and fat mass, we measured serum myostatin in all three diet groups. Normal D decreased serum myostatin significantly relative to no-D (Fig. 3, no-D vs normal-D, p < 0.05, ANOVA with Tukey post-tests). High-D did not further alter serum myostatin relative to normal-D (Fig. 3, normal-D vs high-D, p > 0.05, ANOVA with Tukey post-tests). This first decrease in myostatin (Fig. 3a) occurred without any change in fat free mass (Fig. 2c) suggesting that normal-D decreased average production of myostatin by muscle mass. The unchanged average myostatin in the high-D diet compared to normal-D occurred in the context of increased average lean mass (Fig. 2b), suggesting that high-D further decreased average myostatin production per muscle mass. Overall, these results are consistent with increasing doses of dietary vitamin D inhibiting myostatin production (Fig. 3b).

Normalizing vitamin D improves leptin generation per fat mass, but high-dose vitamin D increases leptin sensitivity

**Figure 4:**
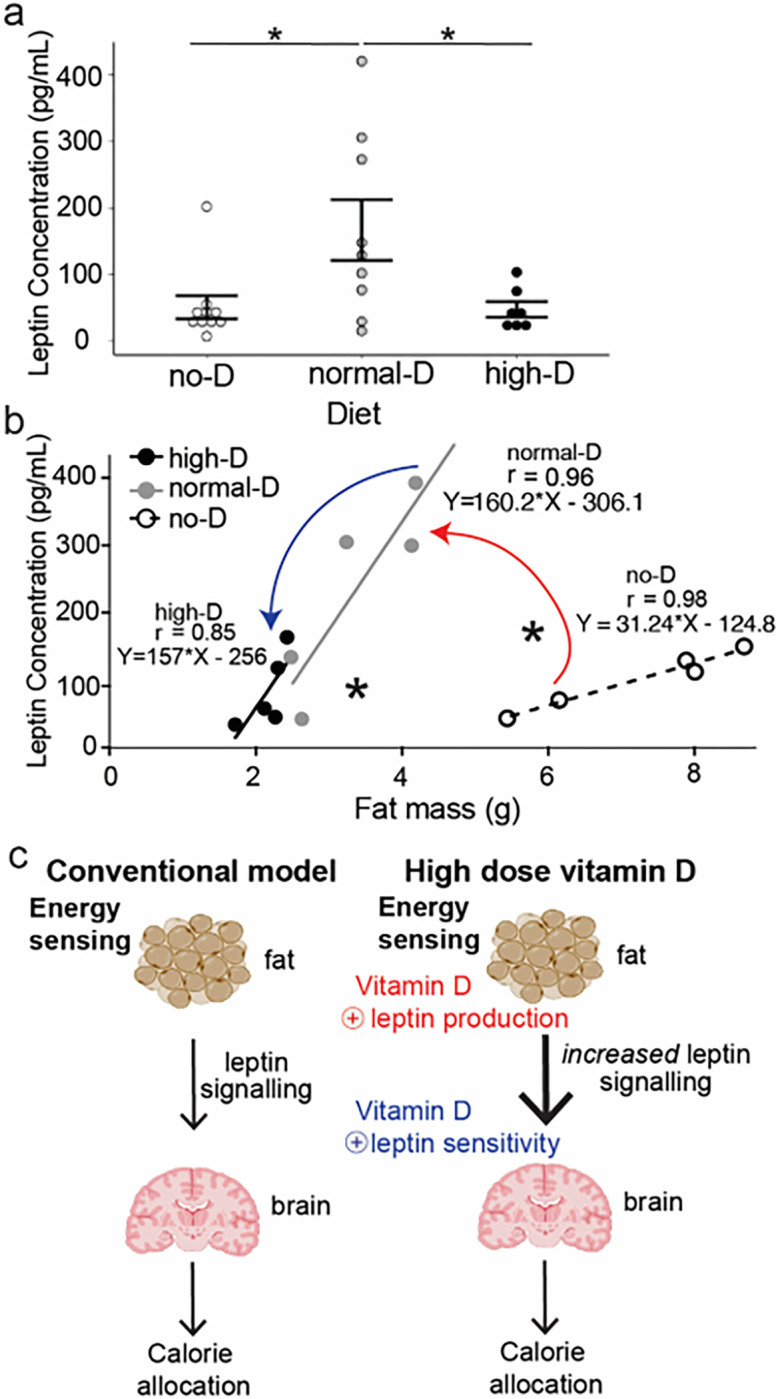
Increasing vitamin D increases leptin production and improves leptin sensitivity. Wild-type mice were maintained on defined CHOW diets with different doses of vitamin D. At sacrifice leptin was measured by ELISA. **a**: Dietary vitamin D alters serum leptin concentration (*= p<0.05 for each ANOVA with Tukey post-tests). **b**: Dietary vitamin D alters serum leptin production per fat mass; the CHOW no-D line slope is different from both the reg-D and high-D lines (*= p<0.05 for each of Normal-D vs no-D, and High-D vs no-D by ANOVA with Tukey post-tests), all three correlations are significant: for no-D: r = 0.98 (F < 0.01), for normal-D: r = 0.96 (F < 0.01) and for high-D: r =0.85 (F < 0.05)). **c.** Normalizing vitamin D increased the amount of leptin produced per fat mass (red arrow in **b,** red text in **c**). Raising D from normal to high shifts the distribution of fat masses (blue arrow in **b,** blue text in **c**) consistent with increased leptin sensitivity.

Vitamin D receptor deficient mice are hypoleptinemic and are largely deficient in white fat, the type of fat largely responsible for leptin secretion. To examine the possibility that vitamin D dose alters leptin signaling to regulate the proportion of muscle mass and fat mass, we measured serum leptin in all three diet groups of wild-type mice. Normal-D increased serum leptin significantly relative to no-D (Fig. 4a, normal-D vs no-D). High-D decreased leptin significantly relative to normal-D (Fig. 4a, high-D vs normal-D). To better understand how this complex pattern related to leptin production and sensitivity, we examined the relationship between serum leptin, fat mass, and vitamin D treatment group using linear regression. Within a vitamin D treatment group, total fat mass determined serum leptin (Fig. 4b, for no-D r = 0.98 (F < 0.01 for slope being significantly different than zero), for normal-D r = 0.96 (F < 0.01 for slope being significantly different than zero) and for no-D r = 0.85 (F < 0.05, for slope being significantly different than zero)). However the slope of this relationship was different between groups (* p < 0.05 for normal-D and high-D vs no-D by ANOVA with Tukey post-tests). This graph reveals how the relationship between leptin and fat mass shifted significantly and meaningfully between groups (Graph in Fig. 4b, schematic in Fig. 4c). The slopes in this graph (Fig. 4b) are representative of leptin produced per fat mass. Raising vitamin D from low to normal significantly increased the slope of the curve for leptin vs fat mass (*red arrow* in Fig. 4b, *red lettering* in Fig. 4c): that is, normalizing vitamin D increased the amount of leptin produced per fat mass (*red arrow* in Fig. 4b). Raising vitamin D from normal-D to high-D did not further change the slope of this relationship. However, increasing vitamin D from normal-D vs high-D shifted the distribution on this line (*blue arrow* in Fig. 4b, *blue lettering* in Fig. 4c). That is, further raising vitamin D from normal to high *did not alter* the slope of the curve for leptin vs fat mass but instead shifted the distribution of fat mass down the curve. This is a shift consistent with high dose vitamin D increasing sensitivity to leptin.

### High dose dietary vitamin D significantly increases energy-expenditure without altering activity level or intake

Previously, another group reported that acute paraventricular hypothalamic injection of 1,25D (the active form of vitamin D) decreases appetite in mice^1^. They interpreted this result to indicate that 1,25D increased leptin sensitivity^1^. While some actions of vitamin D may be acute, most actions of vitamin D are mediated by slower transcriptional changes. Thus, it is not clear that this acute response represents a physiologic effect of vitamin D. Furthermore, leptin has actions on both appetite and energy expenditure, but leptin action is generally thought to alter energy expenditure more than appetite. We wanted to better understand the apparent increase in leptin sensitivity described above (Fig. 4) due to high-dose dietary vitamin D. To further examine this relationship, we measured intake and energy expenditure.

**Figure 5:**
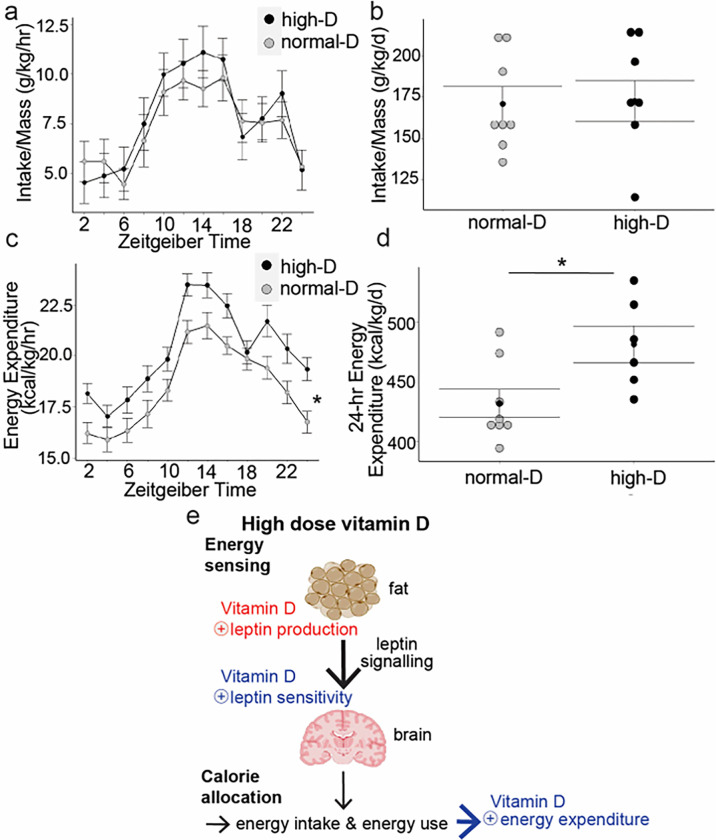
Increasing vitamin D increases energy expenditure without altering intake. Wild-type mice were maintained on defined CHOW diets with different doses of vitamin D. Metabolic cages were used to assay intake, energy expenditure and activity level over one week after acclimatization. **a**, **b**: High dose vitamin D did not alter weight adjusted intake over normal-dose vitamin D. **c**, **d**: High dose vitamin D significantly increased fat-free-mass adjusted 24-energy expenditure relative to normal-dose vitamin D. **e**: High dose vitamin D increases leptin production and sensitivity thus raising energy expenditure.

High dose dietary vitamin D had no effect on mass-adjusted intake (Fig. 5a and Fig. 5b, unadjusted intake was also not significantly different between groups). High dose dietary vitamin D had no effect on activity level (data not shown). However, high dose dietary vitamin D significantly increased fat free mass adjusted energy expenditure (Fig. 5c and 5d, * p < 0.05 by t-test). Raw-energy expenditure (unadjusted for fat free mass) was also significantly different between groups (* p < 0.05 by t-test, data not shown)) without altering activity level. *Thus high-dose dietary vitamin D signals to differentially allocate calories for use by muscle instead of storage as fat without altering weight by increasing leptin secretion and leptin sensitivity*.

### Dietary vitamin D rescues growth factor deficit in the clinic

We replenished dietary vitamin D for 2 months (50000 IU weekly for 4 weeks and 4000 IU/d subsequently), and we repeated these labs (**Table 1**). As we expected, repleting vitamin D normalized serum 25(OH)D and calcium. Surprisingly, repleting vitamin D improved IGF-1 to just above the middle of the normal range for age (z-score 0.05). The initially normal prealbumin and albumin indicated that this young adolescent had appropriate overall energy and protein nutrition and that her low IGF-1 at that time was not related to calorie or protein-calorie availability. In the context of our other data supporting the model that vitamin D conveys energy stores centrally to modulate calorie allocation (Fig. 5e), this result, novelly, suggests that vitamin D also conveys nutrient availability to enable growth.

### High dose dietary vitamin D significantly increases linear growth in mice

**Figure 6:**
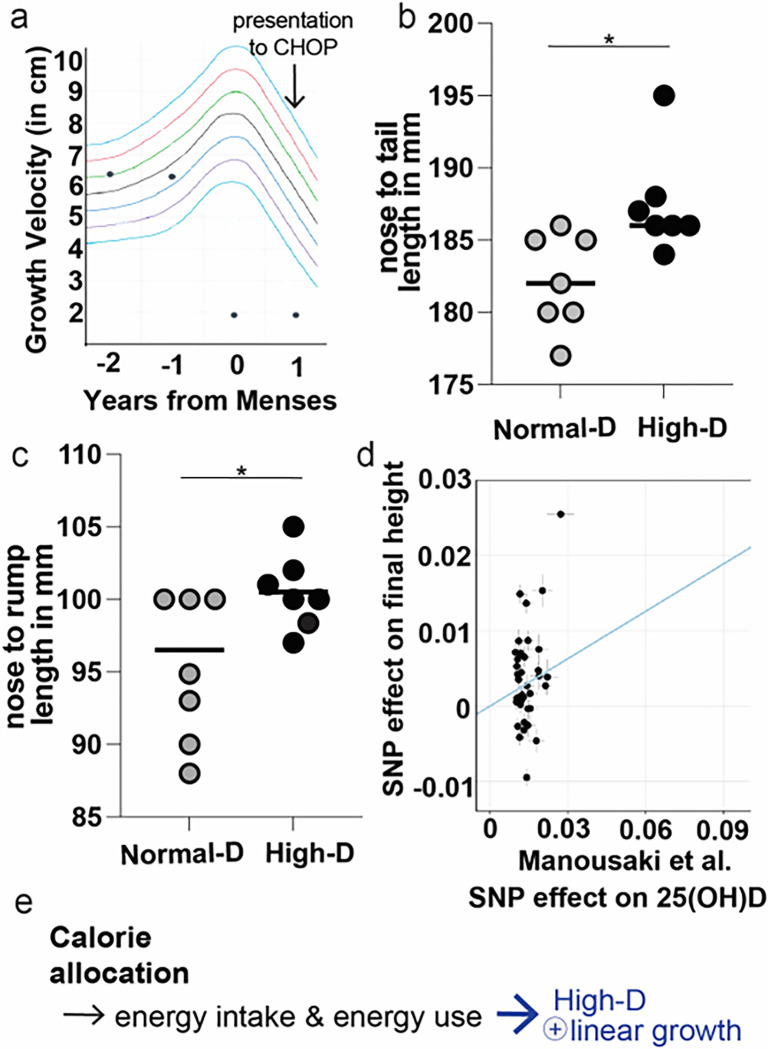
Increasing vitamin D within the normal range increases length. **c**: Vitamin D deficiency caused a premature growth plateau in a single peri-menarchal female that was associated with low IGF-1 (Table 1). IGF-1 recovered with vitamin D supplementation. **b, c:** Wild-type mice were maintained on defined CHOW diets with different doses of vitamin D. Metabolic cages were used to assay intake, energy expenditure and activity level over one week after acclimatization. High dose vitamin D significantly increased length in wild type mice (a: nose to tail, b: nose to rump). **d**: Mendelian randomization reveals a significant relationship between genes that predispose to increased vitamin D and final height. **e**: Schema of how high-D allocates calories to growth.

Our results in mice to this point suggested to us a model where myostatin was not simply playing a homeostatic role at muscle alone, but was also conveying nutrient needs centrally (Fig. 1b for overall model). That is, myostatin conveys not how much energy is being used but what the energy needs are likely to be. If we use the metaphor of the muscles as an engine then myostatin conveys not just how much gas the engine is consuming minute to minute, but how large the engine is. This model would predict that the high-D mediated inhibition of myostatin and increase in leptin signaling would not only increase energy expenditure but would also facilitate increased growth. To examine this possibility, we measured length in anesthetized mice from our diet groups. As we hypothesized based on this model, high-D increased nose-to-tail and nose-to-rump length in mice (Fig. 6b–c; p < 0.05 for each by t-test). This result is consistent with vitamin D facilitating increased central energy sensing across modalities (e.g. for allocation to muscle as well as for use in linear growth).

### A genetic predisposition to higher Vitamin D increases final height in humans

Based on our results in mice, and the interesting result we observed in clinic in an adolescent with early rickets, we theorized that vitamin D might play a similar role in humans to facilitate energy sensing. To examine the possibility that vitamin D might modulate height by regulating calorie allocation in a clinically significant way we wanted to look whether vitamin D influenced height in humans. There are a variety of recent studies that attempt to address this question^18,19^. Interpretation of this work is bedeviled by issues around selection of treatment group (age, vitamin D status, season, etc) as well as duration and dose of treatment. One approach that we have used in other contexts to avoid these issues is Mendelian randomization^20^. We used the most recent genome wide association study (GWAS) to generate instrumental variables for serum 25(OH)D^21^. We applied these results to the most recent and comprehensive GWAS of height^22^. Using this approach we found that our instrumental variables identified significant positive relationships between SNPs that are associated with increased vitamin D and height; (Fig. 6d: Manousakis beta = 0.19, P = 2.09E-3). This result supports the notion that 25(OH)D may influence final height via conveying energy status.

### Raising vitamin D from normal to high-normal in zebrafish embryos increases length

**Figure 7:**
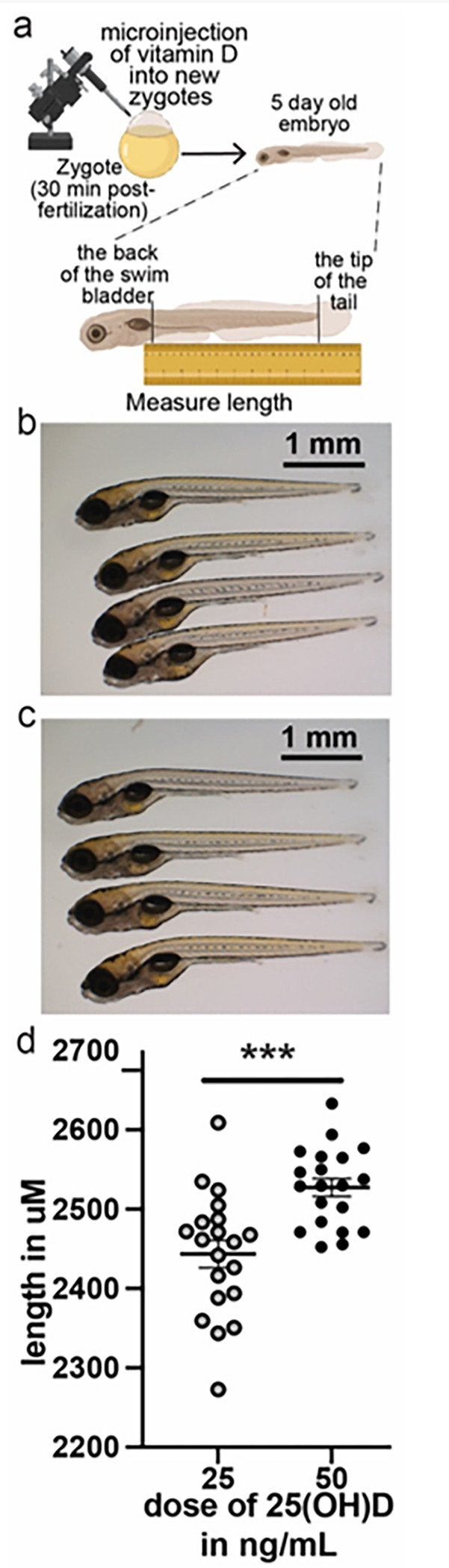
Raising vitamin D to high-normal in zebrafish embryos increases length. **a**: Zebrafish zygotes were injected with vitamin D at fertilization, matured for 5 days and measured, **b**: Typical 5 day embryos injected to achieve 25 ng/mL 25(OH)D, **c**: Typical 5 day embryos injected to achieve 50 ng/mL 25(OH)D, **d:** 50 ng/ml vitamin D significantly increased 5 day embryo length over 25 ng/ml by t-test (*** p < 0.001).

Based on our intriguing results in mice and in humans, we wondered if the role vitamin D plays to convey nutrient status reflects that calories are more plentiful in late summer and fall when vitamin D is generated and stored. This model would predict that the role of vitamin D to convey nutrient status would be evolutionarily conserved in non-mammalian species. To address this hypothesis, we measured growth in zebrafish over the first five days of life (schematic in Fig. 7a) when dosed with normal (25 ng/mL – four typical 5 day old larvae in Fig. 7b) compared with high-normal (50 ng/mL - four typical 5 day old zygotes in Fig. 7c) concentrations of 25(OH)D. We found that high-normal 25(OH)D significantly increased length in 5 day old zebrafish relative to normal (Fig. 7d: 50 ng/mL vs 25 ng/mL **** p < 0.0001 by unpaired t-test, n = 20 for each treatment). Other researchers have previously described that developmental vitamin D deficiency increases fat storage and decreases leanness and length relative to normal vitamin D in zebrafish at older time points. Similar to our results in mice, this result suggests that there are metabolic and growth benefits to raising vitamin D to high-normal relative to low-normal. Overall, these results provide support for the model that 25(OH)D is significantly associated with growth via conveying energy status.

## DISCUSSION

Replenishing vitamin D to normal decreased myostatin production, but further increases of vitamin D did not alter serum myostatin (Fig. 3). Measuring leptin concentrations across dietary groups revealed that replenishment of vitamin D to normal increased the amount of leptin produced per fat mass while high-dose vitamin D increased sensitivity to leptin (Fig. 4). Consistent with the large majority of work on leptin actions, this increased leptin sensitivity did not alter appetite, but did increase fat free mass adjusted energy expenditure (Fig. 5). Thus, here we report for the first time that high dose dietary vitamin D preferentially allocates excess calories to build muscle instead of be stored as fat by increasing leptin production and sensitivity and decreasing myostatin signaling.

In addition, we find that the effect of high-dose vitamin D to increases linear growth (Fig. 6). Using mendelian randomzation we found that the genes that predispose to incrased serum vitamin D (25D) also increase final height. This result confirms the clinical significiance of our work in humans, supporting the model that vitamin D conveys nutrient availability. We also found that high dose vitamin D increases growth in early zebrafish. This effect confirms the evolutionary importance of our work and lends further backing to the model vitamin D conveys nutrient availability.

Strengths of this work include 1) a multidimensional assessment of strength and muscle function simultaneous with metabolism and fat mass, 2) an examination of the effects of low, normal and high-normal vitamin D muscle function and body composition in the context of healthy (e.g. lean) mice, 3) confirmation of the clinical significance of our findings using mendelian randomization and finally, 4) confirmation of the evolutionary significance of our findings using zebrafish. A weakness of this work is that we have not identified each of the mechanistic steps in how vitamin D decreases myostatin signaling and increases leptin production and sensitivity.

**Figure 8:**
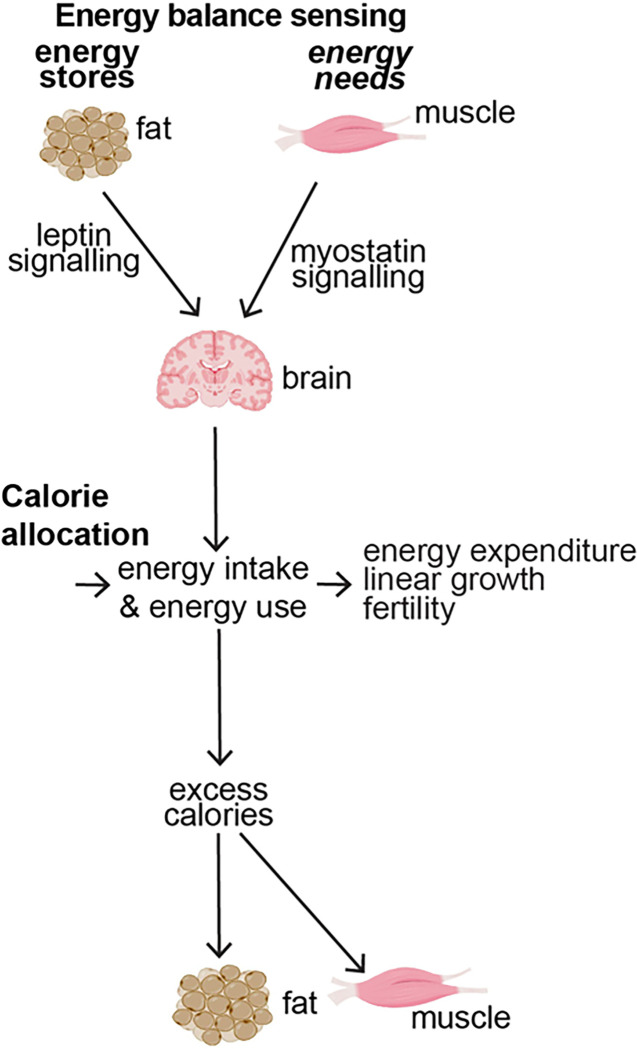
Energy balance sensing. In the (Fig 1a above) energy stores are conveyed by leptin and by default excess calories are stored as fat. in this model, calorie intake can either be used (in energy expenditure) or is excess and thus stored as fat. Our results above (summarized in Fig 1b) reveal roles for vitamin D in modulating energy stores sensing via leptin (Fig 1b.i), energy needs sensing via myostatin (Fig 1b.ii) and calorie allocation to build muscle, for linear growth or for immediate use (Fig 1b.iii–iv). These novel roles give rise to this new model of **Energy balance sensing**. In this new model of there are two critical differences from the old model: first, anticipated energy needs (as conveyed by myostatin) play a critical role in relaying the sufficiency of energy stores, and, second, depending on the sufficiency of energy stores calorie intake will be allocated to linear growth, fertility or to build muscle.

Our results summarized in **Table 2** at left give rise to our proposed new models for vitamin D action (Fig. 1b) and for energy balance sensing (Fig. 8, as opposed to energy stores sensing (Fig. 1a)). In our proposed model raising vitamin D from low to normal increases leptin production by fat, and further raising serum vitamin D levels from normal to high-normal concentrations increases leptin sensitivity (Fig. 1b.i.). Concurrent with these effects, raising vitamin D decreases myostatin signaling (Fig. 1b.ii.). Overall, these changes increase allocation of excess calories to muscle mass (Fig. 1b.iv.) and to linear growth (Fig. 1b.iii.) rather the storage of excess calories as fat.

Notably, these findings for vitamin D give rise to a new model for understanding energy balance (Fig. 8 – a model of energy balance sensing rather than energy stores sensing). In this new model of *Energy balance sensing* there are two critical differences from the old model of energy stores sensing (Fig. 1a); first, anticipated energy needs, as conveyed by myostatin, play a critical role in relaying the sufficiency of energy stores; and, second, in addition to being used as energy expenditure or stored as fat, calorie intake will be allocated also to linear growth, fertility or to build muscle.

A variety of common pathologies including obesity, aging and diseases with chronic inflammation or wasting are associated with decreased muscle function and mass (e.g. sarcopenia). Furthermore, many of these pathologies are associated with low circulating vitamin D because of decreased conversion of calciferols (D2 and D3) to calcidiol^23,24^. Attempts to increase and benefit from an increase in serum Vitamin D have had mixed results in the context of aging, but, recent work in pediatric diseases with chronic inflammation including sickle cell and congenital-HIV identify significant improvements in muscle function with high-dose vitamin D^25,26^. In many diseases with chronic inflammation, weight gain is a proxy for increased health. However, careful assessment of how this weight gain is allocated often reveals that increases in lean mass as opposed to weight in and of itself improve functional measures of disease (e.g. measures of pulmonary function in cystic fibrosis)^27^. Thus, our work provides a physiologic framework for how high-dose vitamin D may be used in these contexts to increase allocation of excess calories to muscle instead of storage as fat. A great deal of work has examined vitamin D effects on weight and BMI with overall equivocal results. There is a small body of prior work suggesting that high dose vitamin D may improve body composition without altering weight or BMI^28–31^.

Several groups have studied leptin as a potential therapeutic for obesity. However, aside from the notable exceptional success in leptin-deficient lipodystrophies, leptin has been largely ineffective in combating obesity in clinical trials^32,33^. The failure of exogenous leptin to effectively treat obesity may reflect that leptin signaling in obesity is past the dynamic portion of the leptin response curve. Nonetheless, the exploration ways to increase leptin signaling or alter leptin sensitivity has been relatively scarce. Our work here provides evidence for the possibility that manipulation of leptin sensitivity can address obesity, and also introduces vitamin D as an initial target for this manipulation. Another possible reason for the failure of exogenous leptin alone as a therapy is that leptin signaling may be only one of several checkpoints in energy balance. The body’s catabolism of muscle once weight loss exceeds 10% suggests that another checkpoint in energy balance may be energy needs. Previously, myostatin has been thought to have homeostatic effects on muscle mass. Our work suggests that myostatin may have an additional role in conveying energy needs centrally. Thus, our work further suggests that addressing energy need signaling by manipulating myostatin pathways can be used to preserve or increase muscle mass in the context of weight loss.

Aside from revealing the role of vitamin D in the interplay between fat and muscle mass, our results are deeply meaningful in at least two other respects. First, these data provide a context for understanding other vitamin D effects beyond its well-recognized function in calcium homeostasis. Viewed through the lens of metabolism, this model provides a chronic or sub-chronic signaling pathway to begin to understand metabolism. This model is a departure from energy stores signaling pathways such as insulin, ghrelin or leptin which convey nutrient status that changes on a day to day or week to week basis. High-dose dietary vitamin D alters the set points of the leptin and myostatin pathways to convey season-specific nutrient availability and use expectations. With this long term signaling that we have identified, calories from a large feast in the winter (when vitamin D is at its nadir) will not be used to increase muscle mass or for a growth spurt but would instead be stored in fat as metabolic insurance against the possibility of scarcity for the remainder of the winter. By contrast, this model would predict that calories from a feast in the late summer or early fall (when vitamin D is peaking) are more likely to be used to improve muscle mass, strength and linear growth.

Second, our work reveals how physiologic seasonal variation in vitamin D may be important in controlling season-specific growth patterns. Humans have long been known to grow more in the summer and fall than the spring and winter. This pattern has been ascribed to historic nutrient availability. However, this pattern has persisted in the developed world where nutrient availability is stable throughout the year. Another possible season specific effect may be on fertility: vitamin D improves fertility in females with polycystic ovarian syndrome (PCOS). PCOS is a disease with abnormal or dysfunctional energy balance signaling. Vitamin D also improves fertility in males. In both of these contexts, vitamin D may also facilitate season-specific fertility.

Overall our work identifies a novel role for vitamin D in nutrient sensing, nutrient needs sensing and calorie allocation (Fig. 1b). This role reveals novel physiology underlying the interplay between fat and muscle and gives rise to a new paradigm for understanding and exploiting control of body composition and calorie allocation (Fig. 8).

## METHODS

### Statistics

Animals were randomized to treatment group by cage. Experimenters were blinded in all experiments to the treatment group of each mouse or zebrafish. All values are presented as Mean ± SEM. Differences were considered statistically significant if p < 0.05 (*) or F < 0.05 (*). For three treatment experiments (e.g. comparisons of low-D, normal-D and high-D) means were compared using a one-way ANOVA with Tukey’s correction for post-tests. For two sample experiments (e.g. comparisons of normal-D vs high-D) a two tailed t-test was used.

### Mouse diets and husbandry

All animal work was reviewed and approved by the Institutional Animal Care and Use Committee of the Children’s Hospital of Philadelphia (Protocol # 0988). To examine calciometabolic independent effects of vitamin D on muscle we used the vitamin D receptor knockout rescue diet (VDRKR diet) and varied vitamin D. We used three levels of vitamin D in the context of defined CHOW VDRKR diets: 0 IU/kg (low – ENVIGO #140078), 2000 IU/kd (normal - ENVIGO #140079) and 10,000 IU/kg (high - ENVIGO #140080). After 4 weeks these diets allowed us to achieve target serum 25(OH)D of less than 5 ng/mL, between 20–30 ng/mL and above 30 ng/mL. The serum calcium, phosphate and PTH were within the normal range in all three groups and were not different between groups. The 1,25(OH)D was undetectable in all three groups. Twelve-week-old male wild-type (WT) C57BL/6J mice (Jackson Laboratories, Bar Harbor, ME) were housed (n = 5 per cage) under a 12:12-h light-dark cycle (light on at 0700) and an ambient temperature of 22°C, and allowed free access to water and diet. Food intake was measured weekly, and body composition was assessed 12 weeks later with nuclear magnetic resonance (NMR) (Echo Medical Systems, Houston, TX).

### Grip strength

Mouse grip strength was measured at the University of Pennsylvania Penn Muscle Institute muscle core with a grip meter (TSE; Bad Hamburg, Germany) as described previously^17^. Briefly, mice were trained to grasp a horizontal metal bar while being pulled by their tail and the force was detected by a sensor. Ten measurements were determined for each mouse and averaged.

### Metabolic assays

Body composition of animals was analyzed by NMR at Mouse Phenotyping, Physiology and Metabolism Core, Perelman School of Medicine, University of Pennsylvania. Then, mice were individually housed in the cages, and their metabolic physiology (intake VO2, VCO2 and RER) was monitored by comprehensive laboratory animal monitoring system (CLAMS) at the Mouse Phenotyping, Physiology and Metabolism Core, Perelman School of Medicine, University of Pennsylvania. Myostatin elisa (Biotechne/R & D Systems Myostatin Quantikine ELISA Kit) and leptin elisa (Biotechne/R & D Systems Mouse/Rat Leptin Quantikine ELISA Kit)were performed on serum samples as per manufacturer instruction in technical triplicate.

### Mendelian Randomization

Mendelian randomization was performed using the TwoSampleMR R package^34,35^. Instruments were identified from exposure GWA studies Manousaki et al. and Revez et al. with a threshold of P = 5E-8^21,36^. Candidate instruments were clumped within 10,000 kb to eliminate LD structure using a 1000 Genomes EUR background (clump_kb = 10000, clump_r2 = 0.001). Outcome data was obtained from Yengo et al^22^. Inverse weighted variance MR was performed using default package parameters.

### Zebrafish Methods

Zebrafish experiments were performed at the Children’s Hospital of Philadelphia Zebrafish core. Zebrafish were injected consecutively within 30 minutes of fertilization with 0.9 nl calcidiol solution to achieve concentrations of 25 ng/mL or 50 ng/mL. 20 larvae for each treatment were imaged in groups of four (4) using a Olympus MVX-10 microscope at five (5) days. Length was measured from distal swim bladder to tail (Fig. 7a) using ImageJ by a blinded experimenter. All procedures using zebrafish were approved by the IACUC of Children’s Hospital of Philadelphia (IAC 001154) and were in accordance with the Guide for the Care and Use of Laboratory Animals (National Academies Press, 2011).

## Data Availability

The datasets generated during and/or analyzed during the current study are available from the corresponding author on reasonable request.
